# Structural and Functional Brain Connectivity of People with Obesity and Prediction of Body Mass Index Using Connectivity

**DOI:** 10.1371/journal.pone.0141376

**Published:** 2015-11-04

**Authors:** Bo-yong Park, Jongbum Seo, Juneho Yi, Hyunjin Park

**Affiliations:** 1 Department of Electronic, Electrical and Computer Engineering, Sungkyunkwan University, Suwon, Korea; 2 Department of Biomedical Engineering, Yonsei University, Wonju, Korea; 3 School of Electronic Electrical Engineering, Sungkyunkwan University, Suwon, Korea; 4 Center for Neuroscience Imaging Research (CNIR), Institute for Basic Science, Suwon, Korea; Leibniz Institute for Neurobiology, GERMANY

## Abstract

Obesity is a medical condition affecting billions of people. Various neuroimaging methods including magnetic resonance imaging (MRI) have been used to obtain information about obesity. We adopted a multi-modal approach combining diffusion tensor imaging (DTI) and resting state functional MRI (rs-fMRI) to incorporate complementary information and thus better investigate the brains of non-healthy weight subjects. The objective of this study was to explore multi-modal neuroimaging and use it to predict a practical clinical score, body mass index (BMI). Connectivity analysis was applied to DTI and rs-fMRI. Significant regions and associated imaging features were identified based on group-wise differences between healthy weight and non-healthy weight subjects. Six DTI-driven connections and 10 rs-fMRI-driven connectivities were identified. DTI-driven connections better reflected group-wise differences than did rs-fMRI-driven connectivity. We predicted BMI values using multi-modal imaging features in a partial least-square regression framework (percent error 15.0%). Our study identified brain regions and imaging features that can adequately explain BMI. We identified potentially good imaging biomarker candidates for obesity-related diseases.

## Introduction

Obesity is a medical condition affecting billions of people worldwide and is considered a serious health issue in the 21^st^ century [1]. Obesity is a key factor for many cardiovascular diseases such as diabetes, hypertension, and stroke [2]. People with obesity have a high body mass index (BMI), a measurement based on weight and height [3]. While BMI is not the most accurate measure of accumulated body fat, it is a convenient and widely adopted measure.

Many studies have adopted neuroimaging tools, including various flavors of magnetic resonance imaging (MRI), single photon emission computed tomography (SPECT), and positron emission tomography (PET), to extract information related to obesity [2,4–6]. MRI is especially useful for assessing both structural and functional brain information. Neuroimaging studies have revealed that specific brain systems are associated with obesity [7–9]. Altered brain activity in brain systems related to reward, motor, cognition, control, and attention has been observed [7]. The reward system has been repeatedly identified as crucial for obesity, and the hypothalamus was shown to play an important role [10–15]. Hormones regulating eating behavior such as leptin and ghrelin convey appetite-related information to the hypothalamus and suppress or promote a person’s food intake [16,17]. Alterations in levels of these hormones confuse the brain’s reward system and might promote weight gain [16,17]. Brain regions controlling the reward system are functionally or structurally related to the hypothalamus and affect food regulation [18].

Multi-modal images of a given brain can be obtained due to recent advances in neuroimaging. Different imaging modalities might provide complementary information and thus lead to better overall information to assess disease-specific changes. We chose two different MRI methods, resting state functional MRI (rs-fMRI) and diffusion tensor imaging (DTI), to explore the brains of non-healthy weight (HW) subjects. Raw data from neuroimaging need to be processed using computer algorithms to extract relevant information [19–23].

One type of analysis algorithm, known as connectivity analysis, focuses on how activities in one region correlate with activities in another region. Connectivity analysis allows observation of the whole brain as a complex interconnected network [24–26]. Resting-state fMRI measures local brain activity using the blood oxygen level-dependent effect (BOLD), while DTI provides *in vivo* neuronal fiber information using anisotropic water diffusion. DTI has several limitations and cannot distinguish between efferent and afferent connections; however, it is the only practical option for assessing *in vivo* fiber information. Connectivity derived from fMRI, referred to as functional connectivity, reflects functional correlations between time series data of different regions. Connectivity derived from DTI is known as structural connectivity, because DTI reflects actual structural connections *via* neuronal fibers between regions.

Previous studies have explored functional brain connectivity of non-HW subjects and have reported decreased connectivity in the right anterior cingulate cortex and left insula, and increased connectivity in the bilateral precuneus, putamen, and posterior cingulate cortex [27,28]. Increased BOLD signals were observed in insula and orbitofrontal cortex [10]. Another study explored structural brain connectivity of non-HW subjects and reported low structural integrity of the connection between frontal and temporal lobes [29]. Cortical volume and thickness reductions were observed in the insula, thalamus, and orbitofrontal cortex [11,13,15]. These studies generally used a single imaging modality to extract connectivity information. In the present study, we used multi-modal imaging to explore connectivity differences between HW and non-HW subjects.

Group-wise differences from connectivity analysis might be used as biomarker of important clinical variables. We attempted to predict BMI values from multi-modal imaging features derived from group-wise connectivity analysis. The aim of this study was to assess differences in connectivity between HW and non-HW subjects using multi-modal neuroimaging.

We obtained rs-fMRI and DTI data from the Human Connectome Project (HCP) database [30]. Connectivity analysis was applied to the following brain regions, which were reported by other studies to be involved in obesity and to be related to the reward system; thalamus, insula, putamen, and orbitofrontal cortex [10–15]. We identified regions that showed group-wise structural connection differences. Structural connections and functional connectivity that was correlated with structural connections were retained for further regression analyses. Regions and associated features were used to predict BMI scores in a partial least-square regression (PLSR) framework. In summary, we identified regions and features from rs-fMRI and DTI responsible for group-wise differences, and used the results to predict BMI values.

## Materials and Methods

### Subjects and imaging data

Institutional Review Board (IRB) of Sungkyunkwan University approved our retrospective study. Our study did not require participants’ consent as we analyzed anonymized data. Participant data were anonymized prior to analysis. We obtained DTI and rs-fMRI data from the HCP database [30]. This study included 120 subjects divided into a healthy weight (HW) group (n = 60) and a non-HW group (n = 60). Groups were classified using BMI scores. All individuals in the HW group had a BMI less than 25; the mean BMI was 22.53 kg/m^2^ with a standard deviation (SD) of 1.37. All individuals in the non-HW group had a BMI greater or equal to 25; the mean BMI was 32.55 kg/m^2^ with a SD of 3.60. BMI scores were significantly different between the two groups (p < 0.05). The mean age of the HW group was 28.93 years (SD 3.48 years), while the mean age of the non-HW group was 29.60 years (SD 3.60 years). The ratio of males and females was 24:36 in the HW group and 25:35 in the non-HW group. Age and gender were not significantly different between the two groups (p > 0.05). No subjects had mental disorders according to the diagnostic and statistical manual of mental disorders fourth edition (DSM-IV) [31].

The following imaging parameters were used for DTI on a Siemens Skyra 3T scanner: image matrix = 168 x 144; pixel resolution = 1.25 mm isotropic; b-value = 3000 s/mm^2^; gradient = 270; b0 images = 18; repetition time (TR) = 5520 ms; echo time (TE) = 89.5 ms; field of view (FOV) = 201x180 mm; flip angle = 78 degrees. For rs-fMRI, the following imaging parameters were used: image matrix = 104 x 90; number of slices = 1200; pixel resolution = 2.0 mm isotropic; slice thickness = 2.0 mm; TR = 720 ms; TE = 33.1 ms; FOV = 208 x 180 mm; flip angle = 52 degrees. HCP rs-fMRI data provided two different phase encodings that differed from those of the conventional phase encoding: “left-to-right” and “right-to-left.” We concatenated the two phase encodings and considered the concatenated data as one-time series data. Each rs-fMRI acquisition took approximately 15 minutes. The HCP team quality checked imaging data, including excessive head movement, as described in [30]. Data used in this study had no outliers in terms of head movement.

### Image pre-processing: DTI

The HCP database provides pre-processed DTI and rs-fMRI data for many commonly performed pre-processing steps using FSL and FreeSurfer software [20,21,32]. Pre-processing steps and procedures applied to analyze the DTI data are described briefly below. The intensity of mean b0 images was normalized across six diffusion series. Echo planar (EPI) distortions were removed using the TOPUP algorithm in FSL. Distortion-corrected images were not different between the two groups. Eddy current-induced distortions and subject motion were corrected with the EDDY algorithm in FSL. All b0 images were registered to T1-weighted images using FSL’s FLIRT algorithm, and the registration results were refined using FreeSurfer’s Bbregister algorithm. Registered images were imported into standard Montreal Neurological Institute (MNI) structural space.

### Image pre-processing: rs-fMRI

The following procedures were applied to the rs-fMRI data: distortions due to gradient non-linearity were corrected with the gradient_nonlin_unwarp package in FreeSurfer. Head motions were corrected by realigning all volumes to the single-band reference image acquired at the beginning of data acquisition using FSL’s FLIRT. Motion-corrected images were co-registered onto T1-weighted structural images using FLIRT in FSL and then FreeSurfer’s Bbregister for fine tuning. Co-registered images were registered onto the standard MNI space with non-linear transformation using FNIRT in FSL. Intensity normalization of total volumes was performed with a whole brain global mean of 10,000. Non-brain extraction was performed by applying the standard MNI brain mask to individual subject spaces. Temporal pre-processing of rs-fMRI data was performed using AFNI software [19]. Nuisance variables, such as six rigid motion parameters and white matter and cerebrospinal contributions were regressed out using the 3dDeconvolve package in AFNI. Band-pass filtering ranging from 0.009–0.08 Hz was applied to remove slow drift using the 3dFourier package in AFNI.

### Tractography and structural network construction

Neuronal fiber densities were extracted to estimate group-wise structural connection differences between the HW and non-HW groups. Many studies have adopted the number of fibers or fiber density to effectively differentiate between healthy and diseased groups, thus we considered fiber density to be a reliable metric [33–37]. Neuronal fiber information was extracted using the Diffusion Toolkit [23]. DTI images were reconstructed with the corresponding gradient tables. Linear least-squares fitting method was used for tensor estimation. Fiber tracking was performed with the Fiber Assignment using the Continuous Tracking (FACT) algorithm, which propagates a line from the center of a seed voxel following the vector orientation, the largest eigenvector, to the next voxel. Tracking was complete when the fiber direction changed rapidly (i.e., angle threshold greater than 35°). A spline filter to smooth tracks and a cutoff filter 20–500 mm in length were applied. Connectivity analysis requires regions of interest (ROIs) to be specified so that correlations among them can be investigated. We transferred information from a pre-defined atlas onto individual subject image spaces to specify the ROIs *via* registration. The Automated Anatomical Labeling (AAL) atlas containing 116 ROIs was adopted [38]. Nodes were assigned to ROIs transferred from the atlas. We computed fiber density values to construct a structural network. Fiber density between two regions is the number of fiber tracks per unit surface and is computed as $\frac{2}{s_{u}+s_{v}}{\sum^{\text{​}}}^{\text{​}}\frac{1}{l\left(f\right)}$, where *S*_*u*_ and *S*_*v*_ denote the cortical surface areas of the two regions involved, respectively, and *l*(*f*) denotes the length of fiber *f* [36]. Edge values were fiber density values that were entered into the matrix as elements. This matrix was referred to as the structural connectivity matrix. The fiber tracking algorithm in this study and many other studies cannot distinguish between efferent and afferent connections [39–41]. We adopted a simple undirected network model. Our tractography method estimated the number of fibers starting from one brain region and ending at the other brain region. Not all brain regions are interconnected, thus we expected that some pairs of regions would not be connected.

### Functional network construction

A functional network was constructed in a similar manner to that used for structural network construction. The same atlas was used for ROIs and network node specification. Each edge value was the Pearson correlation between pre-processed time-series data of two ROIs. Edge values were entered into the functional connectivity matrix, which was further normalized using Fisher’s r-to-z transformation.

### Combining structural and functional connectivity

All subjects had respective structural connectivity matrices, and we searched for connections (i.e., elements in the matrix) in the whole brain that distinguished between HW and non-HW subjects (p < 0.05, corrected). The multiple comparison issue was corrected using permutation tests randomly assigning HW and non-HW cases 5,000 times to identify statistically significant structural connections [42]. Some subjects had no fiber connecting two ROIs, and thus were excluded from the analysis to identify significant connections. Such removal of subjects might cause bias, therefore we confirmed that the proportion of removed subjects was relatively low (i.e., less than 20%). Among the structural connections that differed between groups, we focused on regions in the reward system previously identified as important to obesity research [10–15]: thalamus, insula, putamen, and orbitofrontal cortex regions. The identified structural connections were further analyzed with functional connectivity. A structural connection involves two ROIs, and mean functional connectivity values of both ROI were computed. The mean functional connectivity of a region was computed as the mean of correlation values between the given region and the rest of the brain. For example, a structural connection between region A and region B were first tested with the functional connectivity of region A and then tested with the functional connectivity of region B. We adopted the mean functional connectivity value, as this is a scaled version of degree centrality. Degree centrality quantifies the importance of a given node (i.e., region) in terms of the information flow in a network [24,43,44]. If the structural connection was significantly correlated (p < 0.05) with the functional connectivity of either of the two regions, then the structural connection was retained. We focused on regions whose functional connectivity was correlated with structural connection. For example, if we identified 12 structural connections from DTI, then up to 24 ROIs were considered for functional connectivity. The identified ROIs and the associated features, structural or functional connectivity, were further used to predict BMI values. Strong functional connectivity (via rs-fMRI) does not imply actual structural connection (via DTI). We considered regions with significant functional and structural connectivity in terms of group-wise difference so that a limited number of independent variables would be entered into the regression framework.

### BMI prediction

The identified ROIs and the associated features were used as independent variables in a partial least-square regression (PLSR) framework to predict BMI values [45–47]. First, each independent variable was tested using a simple linear regression model (i.e., one independent variable) in terms of its R-squared value to quantify the contribution of each feature to BMI prediction. Then, PLSR was applied using many independent variables. PLSR is especially useful in a regression framework when there are many independent variables. PLSR is a combination of principal component analysis (PCA) and multiple linear regression [45]. The PCA portion of PLSR ensures the avoidance of the multi-collinearity problem [45]. A major parameter in PLSR is the number of latent variables (LVs). We made sure that we did not overfit the data by determining the number of LVs from the predicted residual estimated sum of squares (PRESS) [45]. The prediction quality of PLSR is measured using PRESS values [45]. The number of LVs was increased until the value of PRESS no longer improved [45,47]. The error value was computed to quantify differences between the predicted BMI and actual BMI. Two types of error, root mean squared (RMS) error and percent error, were computed.

### Statistical tests

Leave-one-subject-out cross validation was applied by assigning 119 (= 120–1) subjects to the training set and the remaining subject to the test set. Differences between HW subjects and non-HW subjects were assessed within the training set using permutation tests randomly assigning HW and non-HW cases 5,000 times to identify statistically significant structural connections (p < 0.05, corrected) [42]. Each identified structural connection was correlated with functional connectivity from rs-fMRI using standard Pearson’s correlation. Structural connectivity of a given ROI was retained only if the correlation was significant (p < 0.05). We quantified the abilities of the chosen ROI and associated features to explain BMI values using R-squared (for simple linear regression) and explained variance (for PLSR) statistics. The explained variance increases as the number of LVs increased. However, we avoided overfitting the data by determining the number of LVs from PRESS. All statistical analyses were performed using MATLAB (Mathworks Inc., USA).

## Results

### Structural connectivity and group-wise difference

**Fig 1:**
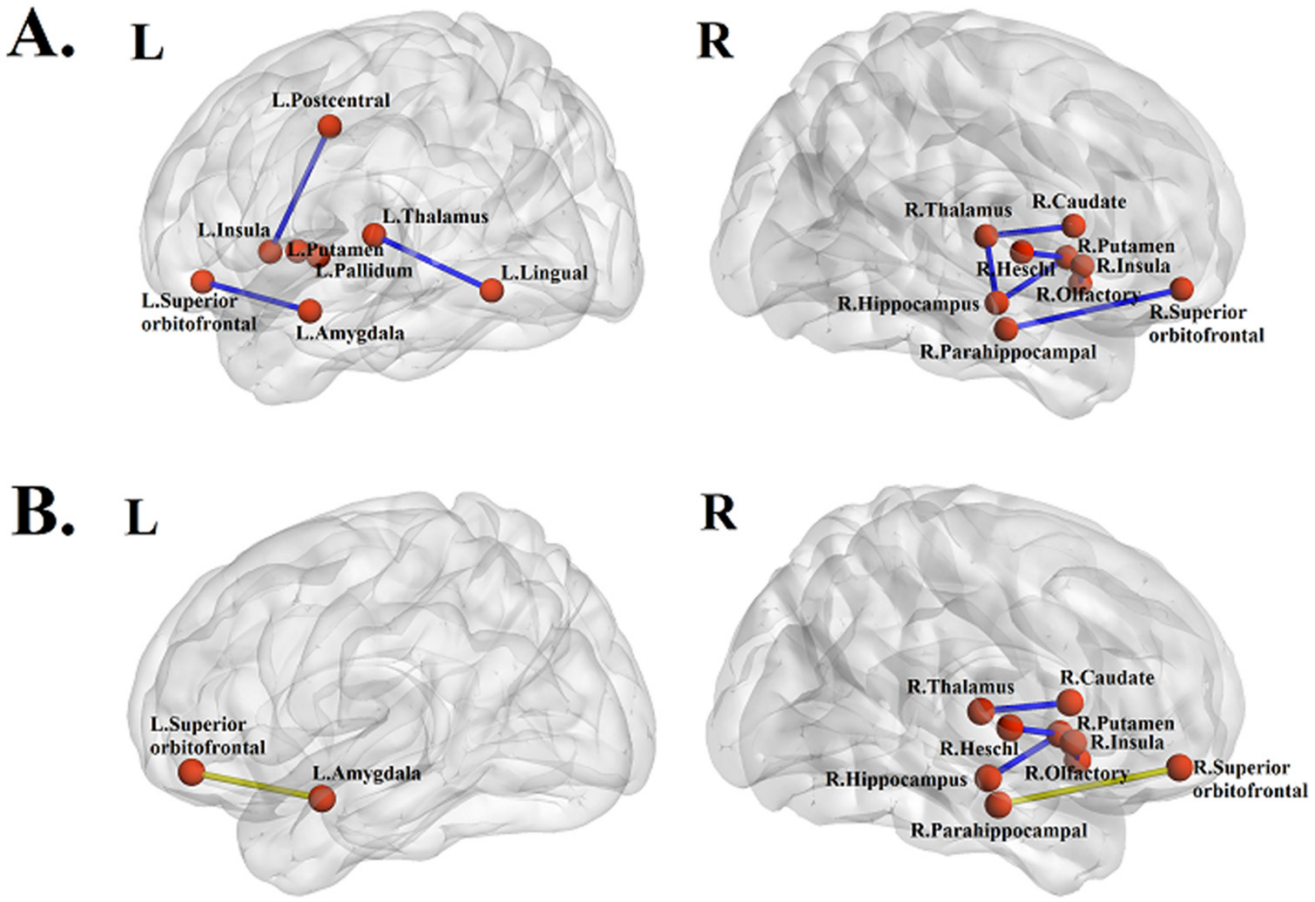
(A) Structural (fiber density) connections with significant group-wise differences. (B) Structural (fiber density) connections that showed significant correlations with fMRI. Green dots and lines represent nodes and edges showing the most significant correlation between structural and functional connectivity, respectively.

Group-wise structural connection differences between HW and non-HW subjects were quantified using leave-one-subject-out cross validation. The distribution of the fiber lengths was given in the supplement (Table A and Figure A in S1 File). The mean length of the fiber was 26.2mm, and it corresponded to the typical length of the neuronal fibers [48,49]. We repeated the process 120 times to identify features with significant group-wise differences. The identified features changed very little among training sets. We report the most representative set of features in this section. We found 10 structural connections showing significant (p < 0.05, corrected) group-wise differences between HW and non-HW subjects (Table 1 and Fig 1A). The regions related to the reward system are the thalamus, insula, putamen, and orbitofrontal cortex. The structural connection between the right medial orbitofrontal cortex and right olfactory had the lowest p-value.

**Table 1:** Structural (fiber density) connections with significant group-wise differences.

Regions	p-value
Left thalamus–Left lingual gyrus	0.0192
Right thalamus–Right Hippocampus	0.0264
Right thalamus–Right caudate	0.0068
Left insula–Left postcentral gyrus	0.0388
Right insula–Right olfactory	0.0476
Left putamen–Left pallidum	0.0070
Right putamen–Right hippocampus	0.0294
Right putamen–Right Heschl gyrus	0.0108
Left superior orbitofrontal cortex–Left amygdala	0.0032
Right superior orbitofrontal cortex–Right parahippocampal	0.0448

#### Combined connectivity and group-wise difference

**Fig 2:**
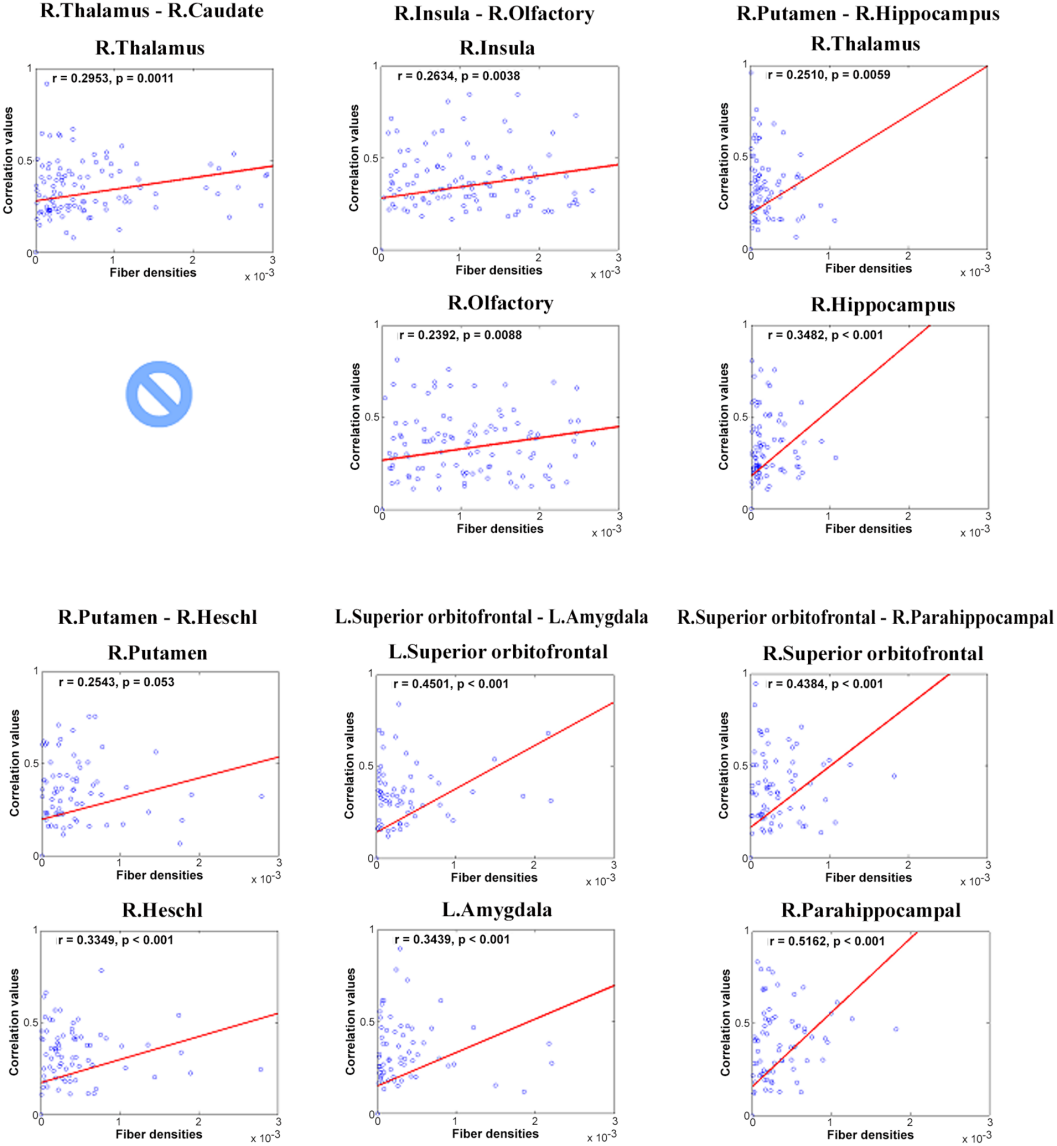
Correlation between structural and functional connectivity of various regions. Six connections with significant correlations between structural and functional connections are reported.

Each of the identified structural connection from the previous step was correlated with functional connectivity of two ROIs, and structural connectivity was retained only if the correlation was significant (p < 0.05). Four structural connections failed to achieve meaningful correlations with rs-fMRI connectivity, and thus were removed. Only six (of 10) structural connections were retained after correlation with functional connectivity information (Table 2 and Fig 1B). All structural connections showed a significant correlation with rs-fMRI except for the connection between the right thalamus and right caudate (Table 2 and Fig 2). The connection between the right superior orbitofrontal cortex and the right parahippocampal showed the highest correlation value. Six structural connections and 10 functional connectivity values were retained. Among 12 possible functional connectivity candidates, one was removed due to poor correlation with structural connectivity, and another were removed because it was a duplicate of a region already identified. In more detail, structural connections involving the right putamen occurred twice (row 3 in Table 2, right putamen and right hippocampus; row 4 in Table 2, right putamen and right Heschl gyrus); thus, we had only one region of functional connectivity from two structural connections. Similar to what we reported in the preceding section, the identified features changed very little from different training sets. We reported the most representative set of features in this section.

**Table 2:** Structural (fiber density) connections that showed significant correlation with fMRI. r_1_ and p_1_ represent the correlation value and p-value of the first region of the connection listed in the leftmost column, respectively. r_2_ and p_2_ represent the correlation value and p-value of the second region of the connection, respectively.

Regions	r_1_	p_1_	r_2_	p_2_
Right thalamus–Right caudate	0.2953	0.0011	-	-
Right insula–Right olfactory	0.2634	0.0038	0.2392	0.0088
Right putamen–Right hippocampus	0.2510	0.0059	0.3482	< 0.001
Right putamen–Right Heschl gyrus	0.2543	0.0053	0.3349	< 0.001
Left superior orbitofrontal cortex–Left amygdala	0.4501	< 0.001	0.3439	< 0.001
Right superior orbitofrontal cortex–Right parahippocampal	0.4384	< 0.001	0.5162	< 0.001

### BMI prediction

**Fig 3:**
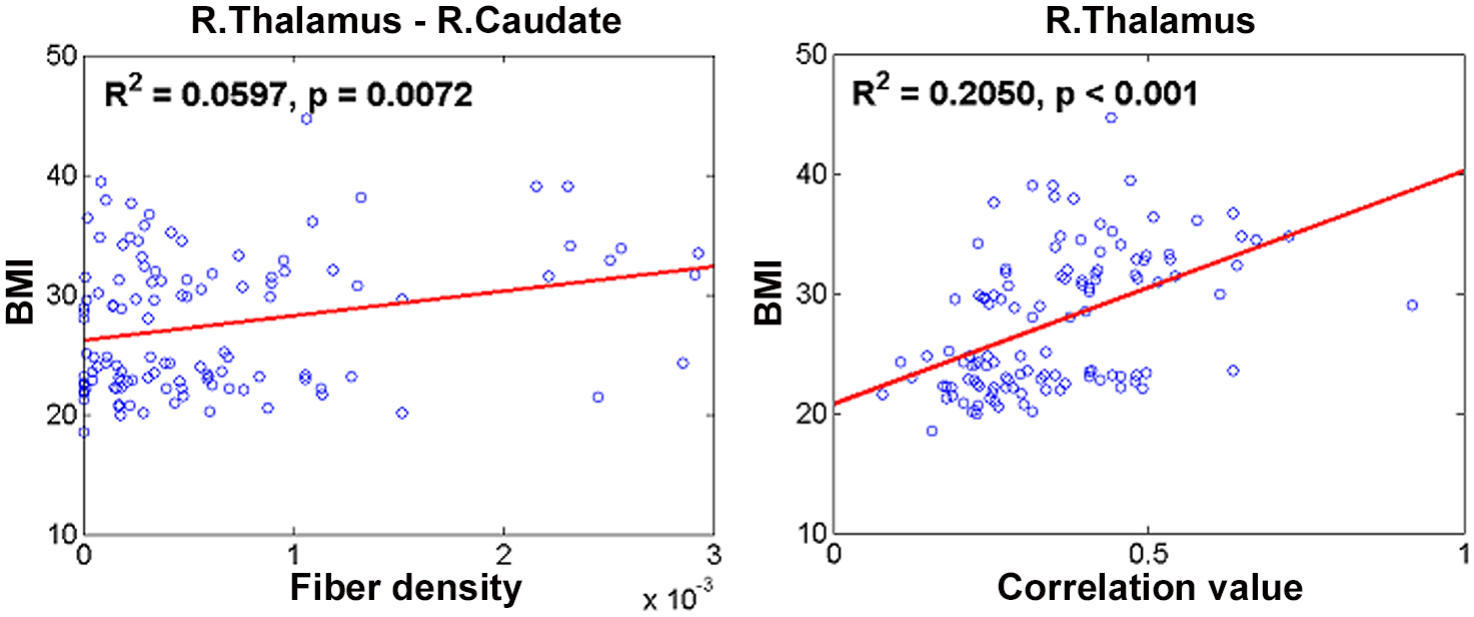
Linear regression results between structural and functional features of different regions that best explained BMI.

The identified regions and the associated features from multi-modal connectivity were used as independent variables in a PLSR framework to determine BMI values using leave-one-subject-out cross validation. The number of LVs in the PLSR framework changed very little for different training sets within the context of leave-one-subject-out cross validation. The most frequently used number of LVs was nine when using all identified regions and features (from both DTI and rs-fMRI), four when using only identified regions and features from DTI, and six when using only identified regions and features from rs-fMRI. Using all identified regions and features (from both DTI and rs-fMRI), the independent variables adequately accounted for BMI values (explained variance = 0.5732). Using only identified regions and features from DTI resulted in an explained variance of 0.3591. Using only identified regions and features from rs-fMRI resulted in an explained variance of 0.3479. Each independent variable was tested using a simple linear regression model (i.e., one independent variable) in terms of R-squared value to quantify the contribution of each feature to BMI (Table 3). Structural connections between the right thalamus and right caudate, right putamen and right Heschl gyrus, and left superior orbitofrontal cortex and left amygdala contributed significantly (p < 0.05) to explaining the BMI values (Table 3 and Fig 3). Among these, the connection between the right thalamus and right caudate was the most significant factor. Functional connectivity of the right superior orbitofrontal cortex and right thalamus contributed significantly (p < 0.05) to explaining the BMI values (Table 3 and Fig 3). Among these, functional connectivity of the right thalamus was the most significant factor.

**Table 3:** Identified regions and features and their contributions to BMI values. R-squared and p-values are reported. Features with two regions are associated with structural (fiber density) connections, and features with one region are associated with functional correlations. Each structural connection has two regions, and the first region is used as the seed region. Structural and functional features that best explained BMI are shown in italicized bold font.

Regions/Features	R-squared	p-value
***Right thalamus–Right caudate***	***0*.*0597***	***0*.*0072***
Left insula–Right olfactory	0.0193	0.1310
Right putamen–Right hippocampus	0.0053	0.4310
***Right putamen–Right Heschl gyrus***	***0*.*0349***	***0*.*0410***
***Left superior orbitofrontal cortex–Left amygdala***	***0*.*0311***	***0*.*0466***
Right superior orbitofrontal cortex–Right parahippocampal	0.0144	0.1910
Left superior orbitofrontal cortex	0.0168	0.1580
***Right superior orbitofrontal cortex***	***0*.*0671***	***0*.*0043***
Right olfactory	0.0235	0.0945
Right insula	0.0271	0.0725
Right hippocampus	0.0044	0.4730
Right parahippocampal	< 0.001	0.9670
Left amygdala	0.0059	0.4050
Right putamen	0.0029	0.5650
***Right thalamus***	***0*.*2050***	***< 0*.*001***
Right Heschl gyrus	< 0.001	0.8780

**Fig 4:**
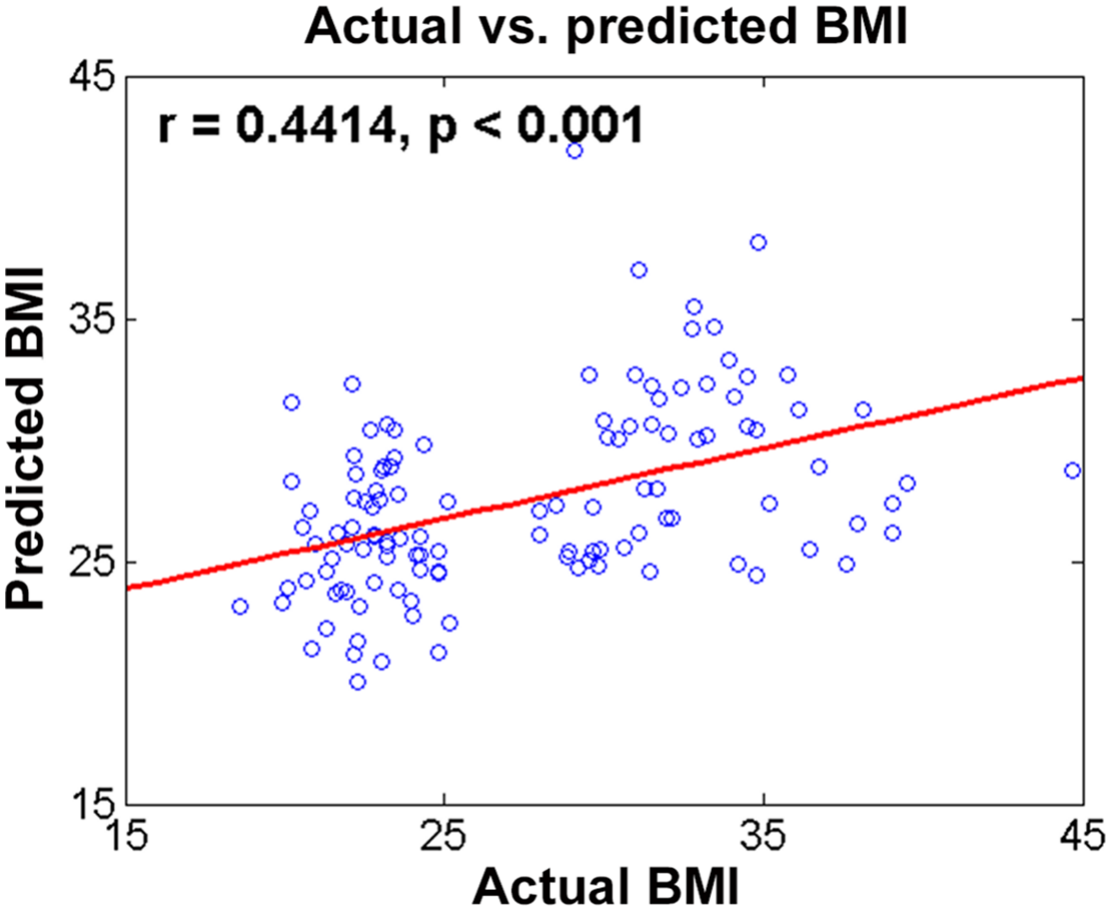
Comparison of actual and predicted BMI between HW and non-HW subjects.

The coefficients of PLSR were computed from the training data, and the model was tested on test data. Predicted BMI values using identified regions and features and actual BMI values showed a meaningful correlation (r = 0.4414, p < 0.001; Fig 4). The mean RMS error between predicted and actual BMI was 5.26 with a SD of 5.26. The percent error mean was 15.0% with a SD of 11.1%.

## Discussion

We considered multi-modal connectivity information from rs-fMRI and DTI to distinguish between HW and non-HW subjects. Significant group-wise differences in structural connections were observed in the thalamus, insula, putamen, and orbitofrontal cortex. Identified connections were correlated with functional connectivity. Statistically significant regions and associated features were used as regressors in a PLSR framework to predict BMI, a widely accepted measure for assessing obesity.

Use of multi-modal features increased the amount of BMI in terms of explained variance (combined 0.5732*vs*. rs-fMRI only 0.3479 and DTI only 0.3591). Rs-fMRI and DTI features complemented each other and better explain BMI. Our BMI prediction model was able to predict BMI values in HW and non-HW subjects. We explicitly avoided overfitting the BMI data by determining LVs of PLSR from PRESS.

Structural connections between the right thalamus and right caudate contributed most to explaining BMI among the structural factors evaluated. This connection was previously identified in research demonstrating that the thalamus and caudate comprise a brain circuit and co-activate on several tasks such as memory and inhibition [50]. Functional connectivity of the right thalamus contributed most to explaining BMI among the functional connectivity-derived factors. Previous studies demonstrated decreased functional connectivity and altered activations in the thalamus in obesity [51–53].

Our study identified brain regions whose structural and functional connectivity measures could predict BMI values well. Obesity is conventionally assessed with body fat percentage, BMI, and other similar measures. Stratifying patients using these conventional measures does not consider the rich neuroimaging data available for many obesity-related diseases, including hypertension and diabetes [2]. Stratifying patients using neuroimaging might allow us to consider brain alterations such as cognitive and executive dysfunctions so that many obesity-related diseases could be better assessed [2,54,55]. Stratifying patients using neuroimaging features (e.g., structural connection between the right thalamus and right caudate within the reward system) could also allow better characterization of obesity-related diseases. The same neuroimaging features could be used to assess the differential impacts of obesity-related hormones such as leptin and ghrelin [16,17]. Further research is needed explore the use of neuroimaging to assess obesity.

We adopted a PLSR framework instead of multiple linear regression to predict BMI values from regressors. We chose PLSR among many possible machine learning-based approaches as we felt that avoiding the multi-collinearity problem among independent variables was important [45–47]. We left exploration of support-vector regression and other machine learning approaches for future studies [56]. Other advanced algorithms exist to analyze structural and functional connectivity. A measure such as betweenness centrality could be used to identify discriminating regions and associated features [26,43]. Two flavors of MRI, DTI and rs-fMRI, were adopted in this study. Several other imaging modalities, including PET, are also sensitive to obesity-related changes [6]. The addition of another modality could provide complementary information and thus lead to better assessment of obesity-related changes.

## Supporting Information

S1 Fig Distribution of fiber lengths. (TIF)

S1 File Distribution of fiber lengths. (DOCX)
