# Supplementary figures and images for: Structural and Functional Brain Connectivity of People with Obesity and Prediction of Body Mass Index Using Connectivity
Source: PLoS One. 2015 Nov 4;10(11):e0141376. doi: 10.1371/journal.pone.0141376 (PMC4633033; doi:10.1371/journal.pone.0141376)

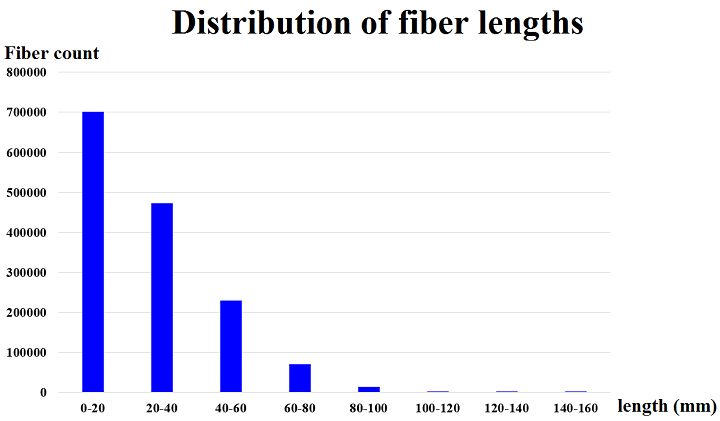

Supplement: S1 Fig — (TIF) [file pone.0141376.s001.tif]
