# Supplementary material for: Structural and Functional Brain Connectivity of People with Obesity and Prediction of Body Mass Index Using Connectivity
Source: PLoS One. 2015 Nov 4;10(11):e0141376. doi: 10.1371/journal.pone.0141376 (PMC4633033; doi:10.1371/journal.pone.0141376)
Supplement: S1 File — (DOCX) [file pone.0141376.s002.docx]

**Supplementary Information**

**S1 Table. Distribution of fiber lengths.**

| **Length (mm)** | **Fiber counts** |
| --- | --- |
| 0-20 | 701,115 |
| 20-40 | 472,418 |
| 40-60 | 229,039 |
| 60-80 | 70,006 |
| 80-100 | 13,204 |
| 100-120 | 2,144 |
| 120-140 | 206 |
| 140-160 | 16 |


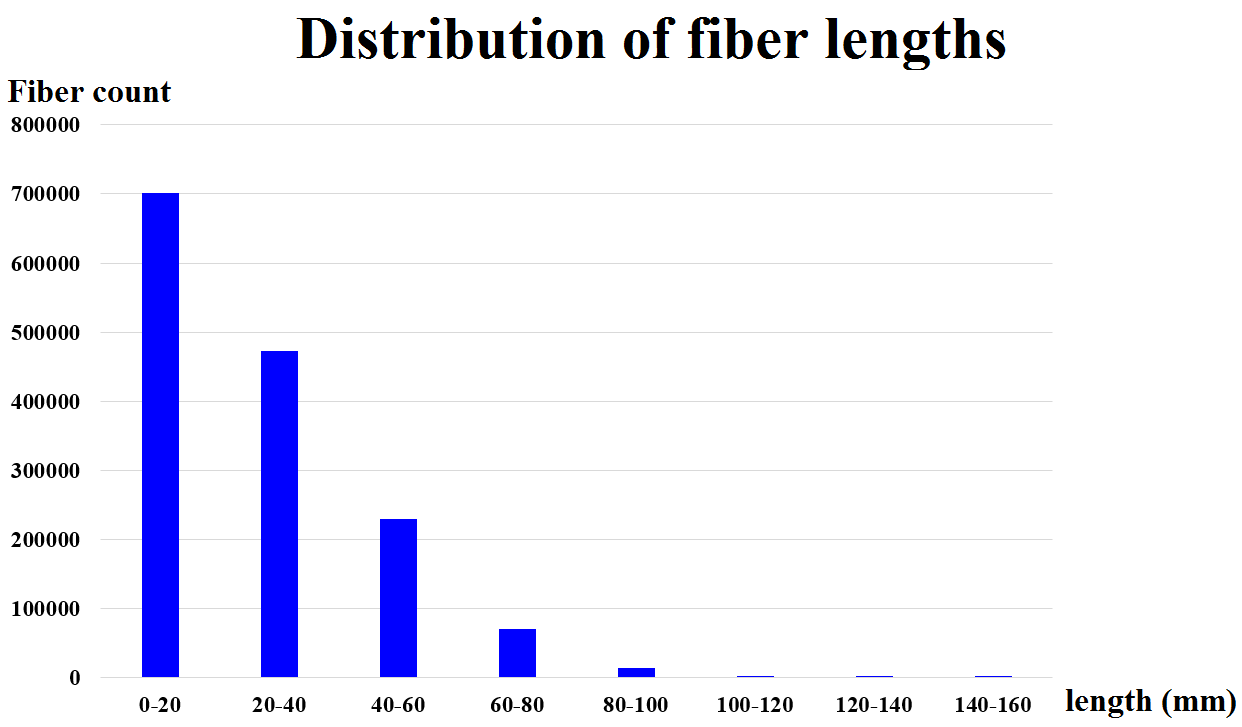


**S1 Fig. Distribution of fiber lengths.**
